# Congruency between publicly available pictorial displays of medial temporal lobe atrophy

**DOI:** 10.1007/s00330-025-11529-w

**Published:** 2025-04-03

**Authors:** Felicia Forseni Flodin, Sven Haller, Leo Poom, David Fällmar

**Affiliations:** 1 Dept of Surgical Sciences, Neuroradiology, Uppsala University, Uppsala, Sweden; 2 CIMC - Centre d’Imagerie Médicale de Cornavin, Genève, Switzerland; 3 Faculty of Medicine, University of Geneva, Geneva, Switzerland; 4 Department of Radiology, Beijing Tiantan Hospital, Capital Medical University, Beijing, China; 5 Tanta University, Faculty of Medicine, Tanta, Egypt; 6 Division of Perception and Cognition, Department of Psychology, Uppsala University, Uppsala, Sweden

**Keywords:** MTA, Dementia, CT, MRI, Brain

## Abstract

**Abstract:**

The medial temporal lobe atrophy (MTA) score is used for visual assessment of MTA on radiological images in suspected neurodegenerative dementia. Although volumetric tools are available, many radiologists still use visual scoring and compare to reference images. Numerous such example images are found online on educational websites and in scientific articles. The aim of this study was to compare congruencies between MTA scores of publicly available sample images with normalized heights and areas of relevant brain structures, measured in the same images.

**Method:**

Systematic online searches yielded 148 individual sample images. The height and area of relevant brain structures were manually delineated, normalized, and compared with regard to the displayed MTA score.

**Results:**

The normalized heights and areas showed correlation with MTA but with considerable overlap between adjacent scores, especially when comparing heights. Also, displays of the MTA score were more consistent with the area of the temporal horn than with the hippocampal area.

**Conclusion:**

There is considerable overlap between adjacent scores in publicly available pictorial displays of the MTA grading system. Insufficient congruency leads to confusion and reduces inter-rater reliability. We also found that publicly available images are more consistent with temporal horn area than the hippocampus, which means that ventricular size may bias the grading. This can impede relevant differential diagnostics, especially regarding normal pressure hydrocephalus. Here, we present lectotype images selected specifically with regard to the hippocampal area.

**Key Points:**

***Question*** *Overlap between publicly available example images of medial temporal atrophy causes confusion and limits reliability.*

***Findings*** *Available images are more consistent with ventricular dilatation than hippocampal atrophy; this article provides lectotype images selected specifically regarding the hippocampal area.*

***Clinical relevance*** *Visual assessment of medial temporal atrophy is used daily and worldwide in radiological examinations regarding suspected dementia. In clinical routine, many radiologists experience uncertainty, and hydrocephalus is often overlooked. This may be caused by insufficient congruency between educational sample images.*

**Graphical Abstract:**

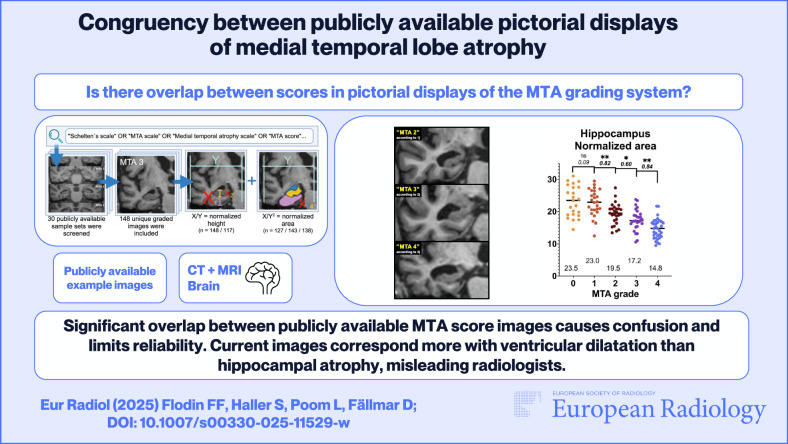

## Introduction

With computer tomography (CT) and magnetic resonance imaging (MRI), it is possible to visualize regional brain atrophy in vivo, which has become an essential part of the clinical work-up of patients with suspected dementia. Particular interest has been directed toward the medial temporal lobe, including the hippocampus [1, 2].

Alzheimer’s disease is the most common type of dementia, characterized by early symptoms of memory loss. The hippocampus and adjacent structures in the medial temporal lobe play crucial roles in memory. Patients with Alzheimer’s disease often exhibit atrophy in these regions, significantly impairing cognitive functions [3, 4].

In 1992 Scheltens and colleagues published an article that defines medial temporal atrophy (MTA) scoring. The study showed that it is possible to visually assess medial temporal lobe atrophy on MR images and distinguish patients with AD from controls [3]. Subsequent studies have shown that a subgroup of AD patients have hippocampal-sparing disease variants [5, 6] and that general atrophy in the aging brain, as well as variations from sex and education level, complicate the MTA assessment [7].

Scheltens’ original study used T1-weighted MR images parallel to the brainstem axis and presented a scoring system from 0 to 4 with increasing atrophy. These scores are based on visual assessment of the height of the hippocampal formation, including the hippocampus (HPC) and the parahippocampal gyrus (PHG), as well as the widening of choroid fissures and temporal horns (TH) [3]. Due to a natural decrease in brain volume with age, some atrophy in the medial temporal lobe is accepted in the elderly, and various cutoff values and age limits have appeared in the literature [8, 9]. The assessment can either be based on an average between the right and left side or a unilateral assessment of the most affected side. Many public sources agree that MTA ≥ 2 is pathological for patients < 75 years and MTA > 2 is pathological in patients ≥ 75 years [10, 11]. In higher age groups, general atrophy has a weaker correlation to cognitive symptoms [12, 13], and some clinical centers emphasize that MTA 3 also can be normal, among the oldest. Some authors suggest using side-averaged values and more fine-grained age groups [9, 14].

Besides the inconsistent cutoff values used for the MTA score, neither the anatomical plane for coronal reconstructions nor the level of coronal slice used have been consistently applied. It has been reported that the appearance varies depending on the reconstruction and that the method needs to be more thoroughly standardized [1, 15].

An important limitation of MTA scoring is the inter-rater agreement; when comparing scores set by different radiologists on the same images. This may be a consequence of vaguely defined landmarks for the coronal slices, as mentioned above, but also because visual grading systems tend to have subjective elements. For example, more attention could either be directed toward the temporal horn or to the hippocampal formation. If too much focus is on the temporal horn, a patient with increased ventricle size (e.g., a patient with normal pressure hydrocephalus and related cognitive impairment), may be misinterpreted as having severe medial temporal atrophy.

Several limitations with MTA scoring were addressed in a study by Cavallin et al that investigated MTA scoring over time [15]. Their inter-rater reliability was “fair to moderate,” and there was a statistically significant difference in scores between raters. The intra-rater reliability was high for an experienced rater but lower for a rater who did not perform MTA scoring daily, with kappa values ranging from 0.38 to 0.74. The authors also stated that inter-rater reliability is lower when two investigators not working together are compared [15]. This conclusion should be seen in light of the worldwide use of the scoring system. Other studies have shown variable inter-rater reliability for the MTA score [8, 16, 17].

From the perspective of cognitive psychology, the MTA score is operationalized so that radiologists estimate length in mm from the images during radiological evaluation. Visual assessment of length is technically a magnitude estimation task where the physical magnitude is assessed by assigning numerical values that reflect the perceived magnitude of the stimulus. It is known that in magnitude estimation of the size of two-dimensional stimuli, perceived size increases more slowly than the physical size, but the relation is not stable and is influenced by various factors [18–20]. For example, subjects spontaneously make use of both height and width when estimating width or height alone [21]. Interestingly, the assessment of length is systematically biased by the area of the object [22].

Our professional experience from teaching and supervising younger colleagues in clinical practice is that radiologists often compare the images of the present patient with publicly available reference images to see which one has the most similar appearance, rather than estimating the actual height or length of a specific brain structure. Therefore, the assessment takes the form of a comparison task rather than an estimation task, and it is plausible that area rather than height is compared. These complex aspects involved in subjective radiological assessments are of important concern regarding the MTA score and other visual assessments and may limit the inter-rater reliability, as mentioned above.

There are many publicly available example images of the MTA scoring system online, usually consisting of five images that represent each MTA score and serve as a reference. Although the MTA score has been successfully used in many well-defined studies, a common experience among radiologists is that MTA scoring in clinical routine is hampered by uncertainty and subjectivity. It is often considered difficult to separate adjacent scores such as MTA 2 and MTA 3, while the impact of this very distinction can have direct effects on the diagnostic work-up.

The first aim of this study was to objectively measure the relevant heights and areas in publicly available pictorial displays of medial temporal atrophy, and compare these with the corresponding MTA scores (as displayed on the sample images) to examine the congruency between them. In line with the perceptual aspects mentioned above, areas were measured in addition to the traditional assessment of height. As a second step, formalized lectotype images for each score were selected from the collection. In biological sciences, a lectotype is a specimen chosen retrospectively to represent a species.

## Materials and methods

### Ethics

The images collected from the Internet are all publicly available and anonymous. It is not possible to trace any of the images to an individual. Thus, ethical application was not necessary for the study. The images were published under Creative Commons licenses, and author approval was obtained for all images reproduced in this study.

### Data collection

Example images of MTA scoring were systematically retrieved from the internet during the winter of 2022. Several selected search words such as “MTA scale,” “MTA score” and “Scheltens scale” were used in the image results function in a web browser to maximize the outcome. During systematic searches, images were copied and collected. Each new result was directly compared to the existing images, and all non-identical images were incrementally added to the collection. The process was repeated many times to achieve search satisfaction and was performed by a last-year medical student under supervision from a board-certified neuroradiologist.

Thirty sets of CT and MR images of brains that displayed MTA scores were identified. The material was checked for duplicates. Brain midline and the full horizontal diameter of at least one temporal lobe were evaluated along with regions of interest. Images, where the intended diameters and areas could not be adequately evaluated due to excessive cropping or insufficient resolution, were excluded. See Supplement 1 for a full list of sources.

After exclusion, a total of 19 sets of images were analyzed, 17 MR sets and 2 CT sets, where the majority contained five examples of respective MTA scores 0–4 on bilateral temporal lobes. Counting both left and right sides, 29 examples were obtained of MTA 0, 28 of MTA 1, 28 of MTA 2, 32 of MTA 3, and 31 of MTA 4. In total, 148 individual images were analyzed. Due to limitations from image cropping and individual differences, not all measurements could be performed on all images. HPC height was measured in all images and hippocampal formation height in 117 images. Areas from TH/HPC/PHG were measured in 127/143/138 images, respectively.

All included images were also categorized by the anatomy shown in the image, representing the actual slice position. The variables were the anterior pons, the crus cerebri, the midbrain and the cerebellar peduncles. Although there is individual variation in anatomy, these landmarks can be used as an orientation for how far anterior the actual image slice is located.

### Measurements

The height of the hippocampal formation (defined as the dentate gyrus, hippocampus proper, and subiculum together with parahippocampal gyrus) was measured in each image according to the original description by Scheltens [1], as well as the height of the hippocampus by itself. This was accomplished by measuring the respective largest vertical heights of both the hippocampal formation and the hippocampus. In addition, areas of the HC, TH, and PHG were manually delineated. All measurements were made using free-hand tools in the open-access image analysis program ImageJ.

**Fig. 1:**
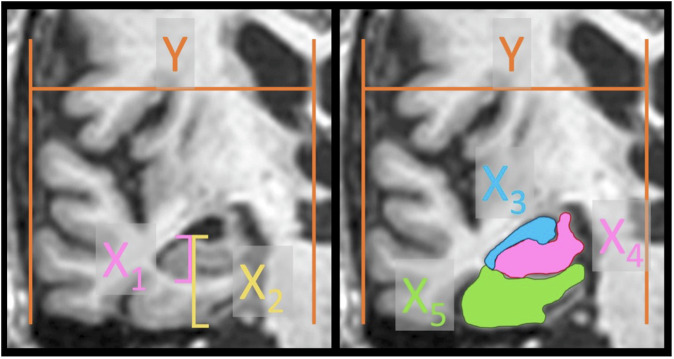
The method for normalized measurements. A reference line was drawn from the midline to the most lateral border of the temporal lobe (Y). The height of the hippocampus (X_1_) and the hippocampal formation (X_2_) was divided by the reference line to get normalized heights for each image. Areas delineating the temporal horn, hippocampus, and parahippocampal gyrus (X_3,4,5_) were normalized by dividing their respective areas by the squared length of the reference line

In the area measurement of the HPC, all but the most distal part, the “tail,” was included in the area. The PHG was delineated using the corner of the collateral sulcus as a lateral end point where a line was drawn straight across the gyrus. The TH was defined as limited by the choroid fissure. The choroid plexus located inside the TH was sometimes visible and used as a marker to confirm the TH space. See Fig. 1 for a representative image.

Since full information about image size and dimensions was not available, it was necessary to normalize the measurements. First, a straight vertical line was drawn through the midline, using the falx cerebri, septum pellucidum, and the center of the pons as landmarks. A line parallel to this was drawn tangentially to the most lateral part of the temporal lobe. Finally, a horizontal line perpendicular to the two prior lines was drawn to measure the distance between them, which was set as a reference line (Fig. 1).

### Calculations and statistics

Each linear measurement (X_1,2_ in Fig. 1) was normalized by dividing by the reference line (Y). Areas (X_3,4,5_ in Fig. 1) were divided by the square of the reference line. The acquired unitless ratio was multiplied by 1000 for practical reasons.

GraphPad Prism (v9.5.1) was used for statistics and graphs. Since results had dissimilar variances and not all categories were normally distributed, non-parametric statistics were used consistently. Spearman correlation coefficients were calculated between each set of normalized heights and areas, versus the MTA scores. Comparisons between results from adjacent MTA scores were calculated with two-tailed, unpaired Mann–Whitney ranked tests.

Each *t*-test was accompanied by an effect size, calculated as a standardized mean difference. For each comparison, the difference in mean values was divided by the standard deviation of the lower group. According to common practice, values of 0.2 to 0.5 were considered a small effect size, 0.5 to 0.8 medium, and > 0.8 were considered large.

## Results

The strongest correlation coefficient between MTA scores and measurements was found for the TH area; 0.892 (*p* < 0.0001, *n* = 127). Correlations between MTA scores and the HPC and PHG areas, respectively, were −0.675 (*p* < 0.0001, *n* = 143), and −0.535 (*p* < 0.0001, *n* = 138). Correlation for height measurements was −0.474 (*p* < 0.0001, *n* = 117) for the hippocampal formation and −0.300 (*p* = 0.001, *n* = 148) for the hippocampus (weakest).

**Fig. 2:**
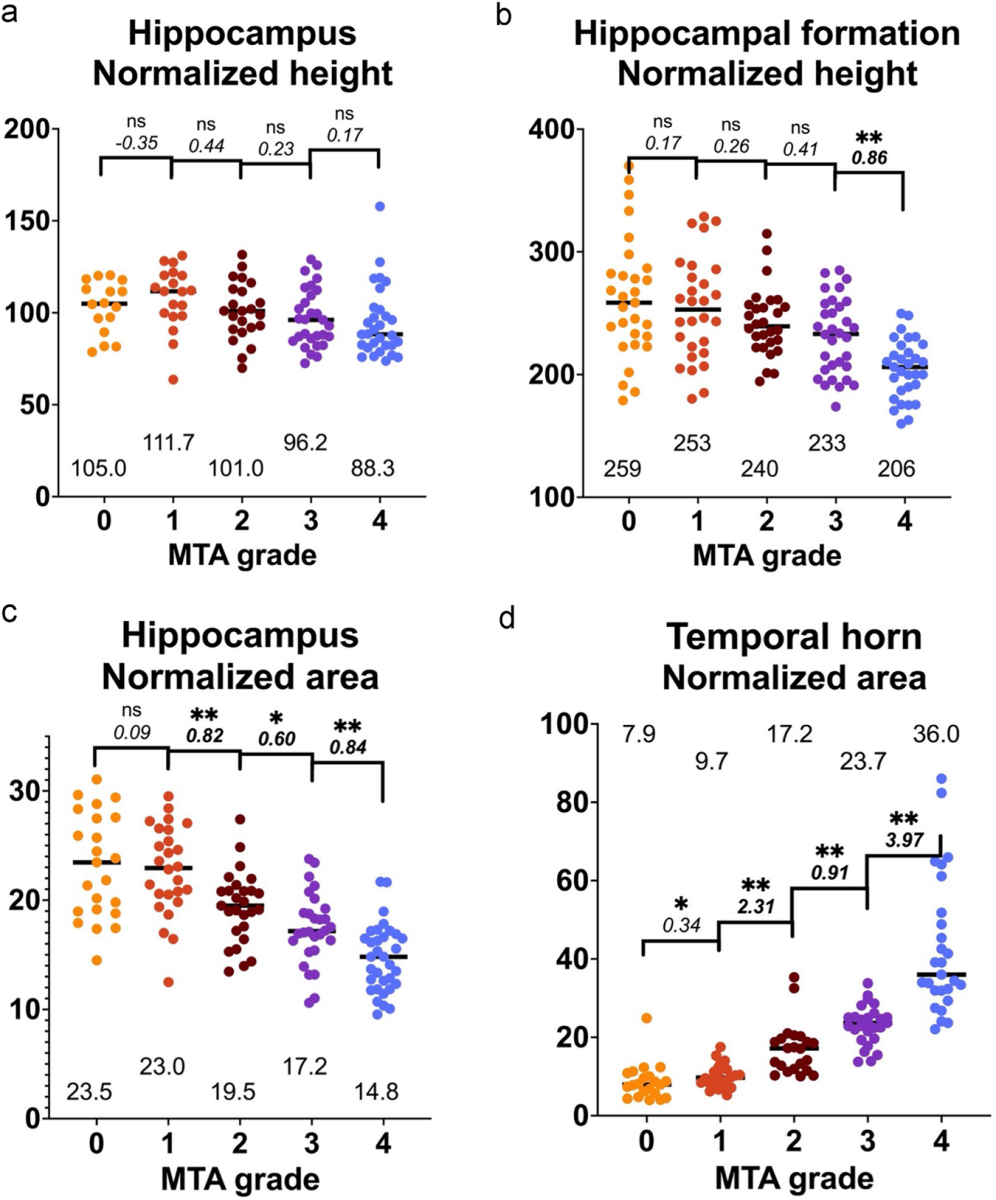
**a**–**d** Grouped scatter plots showing normalized height of hippocampus (**a**) and hippocampal formation (**b**), as well as normalized areas of hippocampus (**c**) and temporal horn (**d**) in relation to MTA scores. Median values with one decimal are shown for each score and marked with a black horizontal line. For each adjacent pair, statistical significance and effect size with two decimals are shown in italics. Effect sizes > 0.80 are highlighted in bold style. “ns” means not significant, “*” means *p*** <** 0.05, and “**” means *p*** <** 0.01

**Fig. 3:**
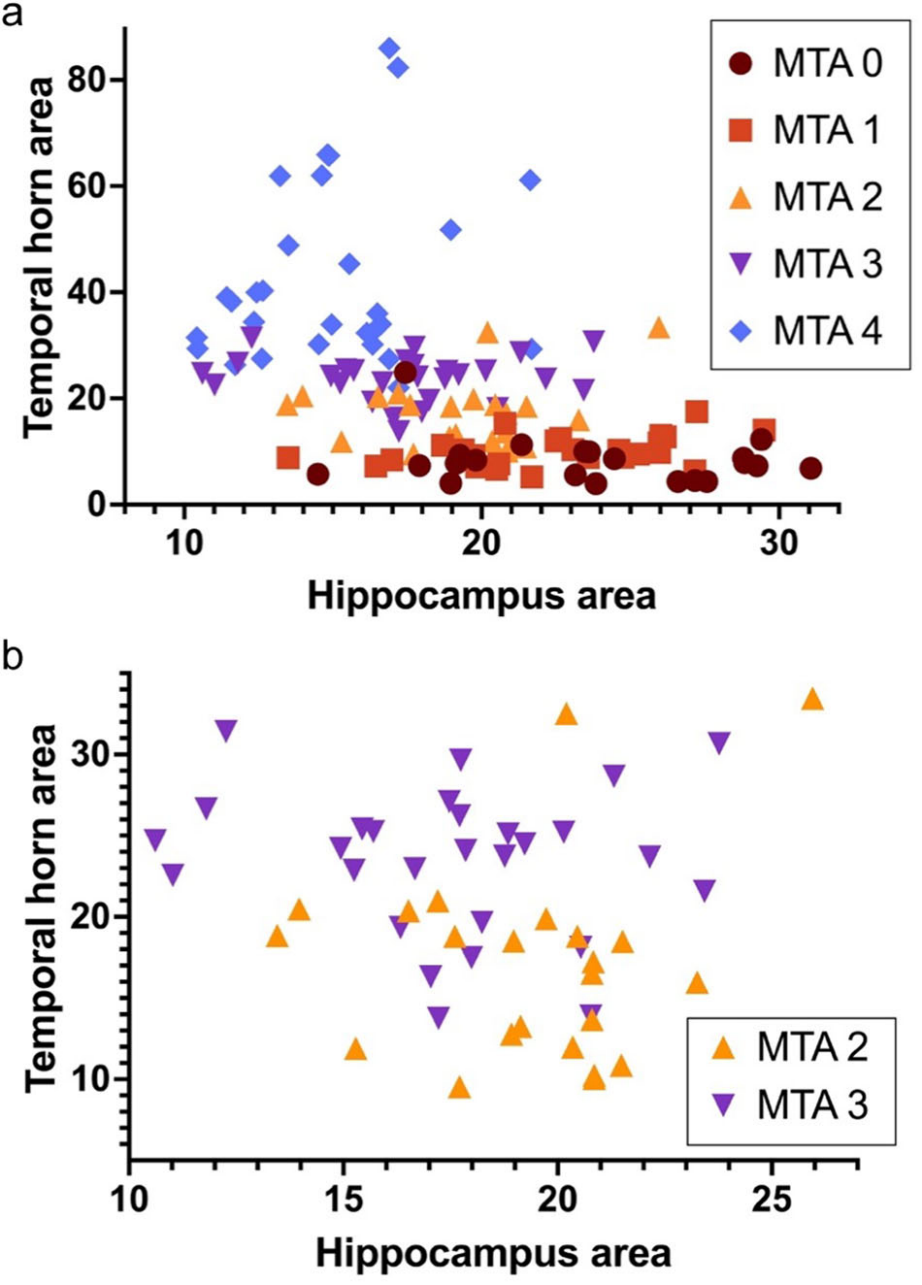
**a**, **b** Scatter plots of normalized hippocampus area and normalized temporal horn area in different MTA grades. In **a**, the most evident exception from the overlap is the larger temporal horn areas in MTA 4. In **b**, MTA grades 2 and 3 are shown exclusively to highlight the overlap in this clinically relevant distinction

Figure 2a–d displays normalized heights and areas for different MTA scores as grouped scatter plots and median values, effect sizes, and statistical significance from *t*-tests between adjacent scores. Results from the PHG area showed similar overlap and variance (data not shown). Figure 3a, b displays scatter plots between normalized hippocampal area and normalized temporal horn area.

**Fig. 4:**
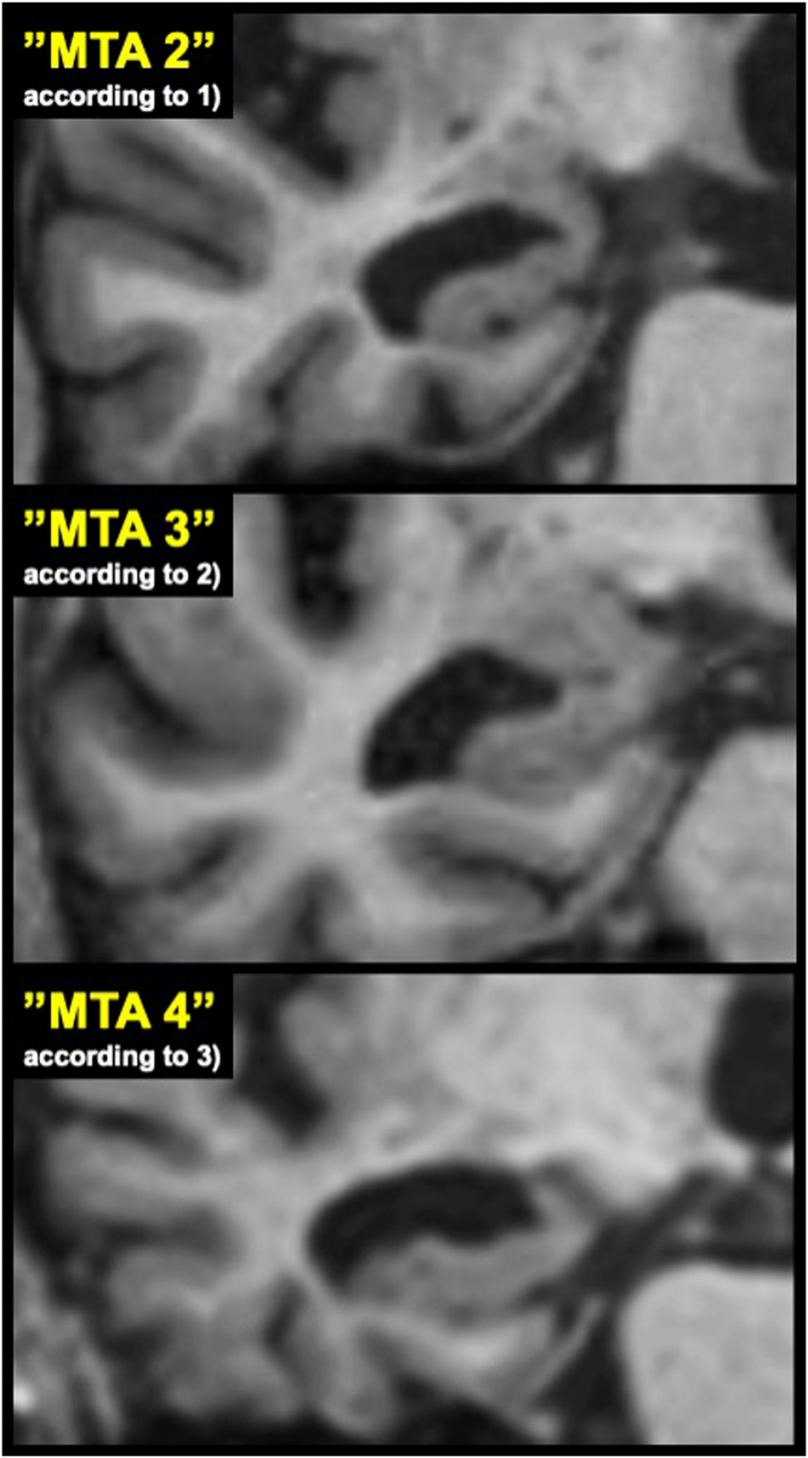
A selection of included sample images of the MTA score that exemplifies the overlap shown in Figs. 2 and 3. These images have a similar appearance but are publicly displayed to represent different steps in the grading scale. The images are reprinted with the permission of the source authors and show coronal T1 images at the level of anterior pons, here cropped to visualize the right medial temporal lobe specifically. Normalized hippocampal areas for these images are 17.6, 18.8, and 21.7 for the top, middle, and lower images, respectively. Normalized hippocampal heights and image sources are listed in Supplement 1

The overlap shown quantitatively in Figs. 2 and 3 is graphically exemplified in Fig. 4, where three selected images have similar visual appearances, despite having been specifically selected to represent different MTA scores.

**Fig. 5:**
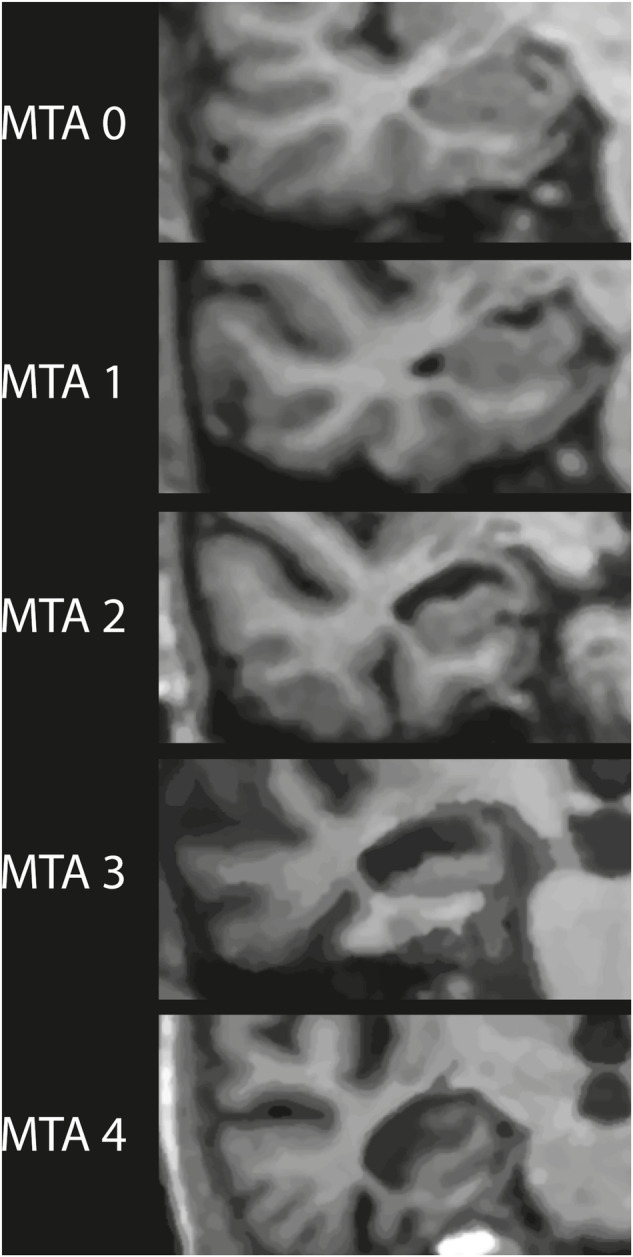
Suggested lectotype images for each MTA grade. They were chosen from the publicly available material included in the study and are reprinted with permission from the source and/or comprised of Creative Commons license. The figure consists of coronal T1 images at the level of anterior pons, here cropped to visualize the right medial temporal lobe specifically. The normalized hippocampal areas for each grade and image area are; MTA 0: 23.46, MTA 1: 23.57, MTA 2: 19.13, MTA 3: 17.54, MTA 4: 14.83. The images have been graphically adjusted with a reduced gray scale range and adapted contrast levels, for generalization and harmonization purposes. Image sources are listed in Supplement 1. Recommendations on cut-off limits vary between sources. Several popular sites currently recommend using a pathological cutoff (on either side) of MTA ≥ 2 for patients < 75 years, and MTA ≥ 3 for patients ≥ 75 years [10, 11]. In the visual assessment, a loss of height in the hippocampal formation should help to separate grade 3 from grade 2. When a large temporal horn is the most prominent finding, radiological features of normal pressure hydrocephalus should be checked

From the total collection of images, representative images were selected that both had an incrementally decreased hippocampal area in close proximity to the median results from the present study, as well as other features consistent with the original descriptions of Scheltens scale [3]. The selected images were post-processed to achieve a standardized and iconographic appearance. These are presented in Fig. 5 as suggested lectotype images for the MTA scoring system and can serve as universal example images between different clinics and research groups.

The positioning of the coronal slice varied between sources, and also between different grades from the same source/author. As shown in Table 1, the most prevalent position was through the anterior pons and with continuously visible crus cerebri (position C). However, there was a clear tendency for displays of MTA 3 and 4 to have more anterior positions.

**Table 1:** The rate of different positions of the coronal plane in the example images

	A	B	C	D	E
MTA 0	9%	19%	47%	25%	0%
MTA 1	0%	35%	48%	16%	0%
MTA 2	7%	33%	43%	10%	7%
MTA 3	16%	41%	22%	19%	3%
MTA 4	9%	43%	23%	20%	6%

A: Image plane more anterior than pons (pons not visible). B: Section through anterior pons, but the crus cerebri are not continuously visible. C: Both anterior pons and continuous crus cerebri are shown. D: Section through middle pons and midbrain. E: Section showing middle/posterior pons and cerebellar peduncles

## Discussion

This study has two main findings. The first is a substantial overlap between publicly available pictorial displays of MTA scores when objectively measuring relevant brain structures. Specifically, the overlap highlights the need for clear distinctions between adjacent grades that currently serve as clinical cutoffs (1 vs 2 in younger patients, 2 vs 3 in older). If the different sample images had been congruent, little or no overlap had been found—i.e., clear distinctions between grades. In the present study, areas (more closely related to volumes) had higher congruency between scores than height (and the height of the hippocampus had the weakest correlation coefficient of all measured structures). However, exact areas cannot be assessed visually, and manual delineation is usually considered too time-consuming for clinical routine. This discrepancy suggests that the limitations inherent in visual grading scales may be an incentive for adapting volumetric tools. Another discrepancy is the insufficient congruency between the tilt/position of the coronal slice, shown in Table 1. The most prevalent position was chosen in less than half of the cases, and images displaying MTA grade 4 had a particularly large variance in positioning.

In the present clinical routine, insufficient congruency between different examples of the same scale leads to confusion and inconsistent results. The overlap in appearance presented in this article (quantitatively in Figs. 2, 3 and Table 1, and qualitatively in Fig. 4) highlights a source of such confusion. Group-level measures of effect sizes ranged from low to fairly high. The individual data points displayed in Figs. 2, 3 offer a visual overview of the overlap, which we consider to be substantial and problematic. That is, many individual data points representing a specific height or area are currently consistent with several different MTA grades, even between groups with larger effect sizes. For the reasons listed above, suggested lectotype images for each MTA score are presented (Fig. 5), with images selected from the present material to match the quantitative analysis.

The second main finding of the study was that the publicly displayed MTA scores are more strongly associated with the area of the temporal horn than with the hippocampus. Also, the TH area had less overlap than HPC measurements, as shown in Fig. 2d and 3a. This may influence radiologists to set MTA scores based more on ventricular dilatation rather than hippocampal atrophy. This can pose a clinical problem in itself, especially in patients with hydrocephalic features, leading to an exaggeration of actual medial temporal atrophy. Early stages of idiopathic normal pressure hydrocephalus can present with memory loss and is an important and treatable differential diagnosis in patients with cognitive issues. Specifically, an overestimation of the MTA grade can lead to a false diagnosis of Alzheimer’s disease in a patient with hydrocephalus, and the opportunity to consider shunt treatment may be lost. Further, a plausible explanation for the large variance found in the TH area in MTA 4 (Fig. 2d, rightmost group) is that some of the patients displayed in the public MTA scales may actually have, to some extent, hydrocephalic features rather than being typical examples of medial temporal lobe atrophy. Since normal pressure hydrocephalus is a common and important differential diagnosis in cognitively impaired patients, the finding of large temporal horns should trigger an assessment of other associated features, such as dilatation of Sylvian fissures, compression of parafalcine sulci, and a narrow callosal angle.

In the assessment of MTA, the hippocampal formation is assessed in conjunction with the choroid fissure and temporal horn—where atrophy of the former leads to secondary dilatation of the two latter. The hippocampus is a key structure for several cognitive domains and is a central locus for the pathology in patients with the most common (limbic-predominant) subtype of Alzheimer’s disease or LATE disease. As described in the introduction, it should be stressed that human perception is not ideally suited for estimating a volume from a two-dimensional image and that diameters influence our assessment. One of the key features separating grades 2 and 3—which is often a clinically relevant cutoff—is a loss of height in the hippocampal formation. This is not clearly seen in many of the publicly available sample images and may be more evident in the provided lectotype selection.

The area of the parahippocampal gyrus was also analyzed. This structure has received less attention than the hippocampus but is still an important structure in radiological assessments of cognitive impairment [23]. In the present study, these results did not provide additive information and were largely omitted from the results section.

In this study, we included both MR and CT images for our measurements, which did not pose any difficulties. MR images were slightly easier to measure accurately due to higher resolution and the superior ability of MRI to visualize structures. However, MTA scoring is considered sufficient for CT and is frequently used clinically with CT scans. Since only a minority of the available images were from CT scans, MR images were selected for figures and lectotype images.

When using subjective visual assessment as a semi-quantitative measurement, as the MTA score, the operationalized procedures should be strictly followed. Any deviations from the methodology, in combination with individual/subjective differences, will reduce the inter-rater reliability of that measurement. This is exemplified by a recent study that found large variations in performance between different formats of display of radiological images, and also between individuals within each format [24]. With specific regard to the Scheltens scale, visual assessments of height/length are influenced by a variety of biases, such as an inability to dismiss width [21] and area [22].

### Limitations

The collection of images evaluated in this study are from different sources, with various image quality and context—with the common denominator that they have all been selected as typical examples for their respective MTA grade by an expert or researcher in the field. Since this comes with innate diversity, the ensuing congruency is limited, both in terms of the users and for this study. Some of the included images had a suboptimal resolution for the exact delineation of the regions of interest. All measurements in the study have been made by a single person which always entails a risk of bias. To minimize the risk of measuring bias, some images and measurements were repeatedly controlled by a senior consultant in neuroradiology (DF) during the measuring process using a feedback system to the assessor (FFF) to prevent a gradual shift in measurements. Hypothetically, severe atrophy may decrease the total width of the brain (here used as a reference line), but the actual impact of this on our results was considered negligible and most likely limited to the MTA 4 group.

A large number of studies have explored the performance of MTA grading in various clinical and research settings, as outlined in the systematic review of Park et al [8]. Although the lectotype images provided here are directly or indirectly chosen as typical examples from such studies, the accuracy and reliability of the resulting selection should ideally be evaluated. Optimally, such evaluations should be performed by independent research groups in different settings. Also, the subjective preference of different decision support images could be explored to strive for more homogenous compliance among radiologists.

Last but not least, it is plausible that some of the images included in this study had been asymmetrically scaled between *X* and *Y*-axes before publication, which could affect both the heights and areas. While this is not improbable, it does not change or diminish our conclusion regarding the confusion that arises for the end users, which is caused by the overlap in visual appearances and lack of congruency.

## Conclusion

First, publicly available displays of MTA scores show considerable overlap, especially regarding the height and area of the hippocampus. The overlap between adjacent grades is problematic since they are used as clinical cutoffs. Consequentially, the inter-rater reliability and robustness of visual MTA grading as a worldwide clinical tool are limited. Consensus use of a single set of well-defined instructional images would be favorable to maintain and optimize the inter-rater reliability of this widely spread scoring system. In this article, selected lectotype images are provided for that specific intention. However, the limitations of visual grading scales may also be considered as an incentive for adapting volumetric tools.

Second, the MTA scores in publicly available images are more consistent with the area of the temporal horn than the hippocampus. This may influence radiologists to set scores based more on ventricular dilatation rather than hippocampal atrophy—which can interfere with efficient differential diagnostics, especially with regard to normal pressure hydrocephalus.

## Supplementary information

ELECTRONIC SUPPLEMENTARY MATERIAL

## Abbreviations

AD: Alzheimer’s disease
CT: Computed tomography
HPC: Hippocampus
MRI: Magnetic resonance imaging
MTA: Medial temporal atrophy
PHG: Parahippocampal gyrus
TH: Temporal horn
