# Supplementary material for: Congruency between publicly available pictorial displays of medial temporal lobe atrophy
Source: Eur Radiol. 2025 Apr 3;35(10):5944–53. doi: 10.1007/s00330-025-11529-w (PMC12417228; doi:10.1007/s00330-025-11529-w)
Supplement: Supplementary file 1 — ELECTRONIC SUPPLEMENTARY MATERIAL [file 330_2025_11529_MOESM1_ESM.pdf]

Congruency between publicly available pictorial displays of medial temporal lobe atrophy.

ELECTRONIC SUPPLEMENTARY MATERIAL

Supplement 1

List of internet sources of collected images:

Image A [1].

Image B [2].

Image C [3].

Image D [4].

Image E [5].

Image F [6].

Image G [7].

Image H [8].

Image I [9].

Image J [10].

Image K [11].

Image L [12].

Image M [13].

Image N [14].

Image O [15].

Image P [16].

Image Q [17].

Image R [18].

Image S [19].

Image T [20].

Image U [21].

Image V [22].

Image W [23].

Image X [24].

Image Y [25].

Image Z [26].

Image A.1 [27].

Image A.2 [28].

Image O.1 [29]

Additional information to Figure 4:

Image sources ; 1: [5, 6, 30]

<u>*Normalized measurements for the selected images*</u>

1)Hippocampus height: 93. Hippocampus area: 17,6.

2)Hippocampus height: 88. Hippocampus area: 18,8.

3)Hippocampus height 98. Hippocampus area: 21,7.

Additional information to Figure 5:

List of normalized hippocampal area and sources of lectotype images:

MTA 0: HPC-area 23.46*,[5]*

MTA 1: HPC-area 23.57*, [5]*

MTA 2: HPC-area 19.13*, [5]*

MTA 3: HPC-area 17.54*, [31]*

MTA 4: HPC-area 14.83*, [16]*

Reference list

1. Gaillard F Medial temporal lobe atrophy score | Radiology Reference Article | Radiopaedia.org. In: Radiopaedia. https://radiopaedia.org/articles/medial-temporal-lobe-atrophy-score. Accessed Oct 2022

2. Fig. 1 Image examples from MTA grades according to Scheltens' ordinal... In: ResearchGate. https://www.researchgate.net/figure/mage-examples-from-MTA-grades-according-to-Scheltens-ordinal-5-point-MTA-scale-CT-to_fig1_353586267. Accessed Oct 2022

3. Westen DV, Torisson G, Londos E, et al (2014) Medial temporal lobe atrophy is underreported in medical inpatients and predicts mortality. In: ECR 2014 EPOS. https://epos.myesr.org/poster/esr/ecr2014/C-2077. Accessed Oct 2022

4. Severity of medial temporal lobe atrophy, as determined using... In: ResearchGate. https://www.researchgate.net/figure/Severity-of-medial-temporal-lobe-atrophy-as-determined-using-Scheltens-Scale-score_fig4_229075740. Accessed Oct 2022

5. Velickaite V, Ferreira D, Cavallin L, et al (2018) Medial temporal lobe atrophy ratings in a large 75-year-old population-based cohort: gender-corrected and education-corrected normative data. Eur Radiol 28:1739–1747. https://doi.org/10.1007/s00330-017-5103-6. Accessed Oct 2022

6. Kang DW, Lim HK (2018) Current Knowledge and Clinical Application of Brain Imaging in Alzheimer's Disease. In: Journal of Korean Neuropsychiatric Association. p 12. https://jknpa.org/ViewImage.php?Type=F&aid=587966&id=F1&afn=55_JKNA_57_1_12&fn=_0055JKNA. Accessed Oct 2022

7. Sarria-Estrada S, Acevedo C, Mitjana R, et al (2015) Reproducibility of qualitative assessments of temporal lobe atrophy in MRI studies. Radiol Engl Ed 57:225–228. https://doi.org/10.1016/j.rxeng.2014.04.007. Accessed Oct 2022

8. Fig. 1. Evaluation scheme for magnetic resonance imaging.... In: ResearchGate. https://www.researchgate.net/figure/Evaluation-scheme-for-magnetic-resonance-imaging-Representative-T1-structural-MR-images_fig1_334618150. Accessed Oct 2022

9. Figure 1: Visual rating scale. Image depicting four degrees of atrophy... In: ResearchGate. https://www.researchgate.net/figure/sual-rating-scale-Image-depicting-four-degrees-of-atrophy-in-Hippocampus-and-Entorhinal_fig1_257072384. Accessed Oct 2022

10. Figure 1: Subtypes of AD based on patterns of brain atrophy from visual rating scales. | Scientific Reports. https://www.nature.com/articles/srep46263/figures/1. Accessed Oct 2022

11. Harper L, Barkhof F, Fox NC, Schott JM (2015) Using visual rating to diagnose dementia: a critical evaluation of MRI atrophy scales. J Neurol Neurosurg Psychiatry 86:1225–1233. https://doi.org/10.1136/jnnp-2014-310090. Accessed Oct 2022

12. Molinder A, Ziegelitz D, Maier SE, Eckerström C (2021) Validity and reliability of the medial temporal lobe atrophy scale in a memory clinic population. BMC Neurol 21:289. https://doi.org/10.1186/s12883-021-02325-2. Accessed Oct 2022

13. Poh T (2012) Role of MRI in diagnosis of mild cognitive impairment, and Alzheimer's disease. In: Brain Stories. https://teddybrain.wordpress.com/2012/12/31/role-of-mri-in-diagnosis-of-mild-cognitive-impairment-and-alzheimers-disease/. Accessed Oct 2022

14. Laczó J, Hort J, Vyhnálek M (2021) Evaluation of brain atrophy using visual rating scales and their clinical contribution to the early and differential diagnosis of dementia. Neurol Praxi 22:358–374. https://doi.org/10.36290/neu.2021.026. Accessed Oct 2022

15. Fig. 1. Sheletens Medial Temporal Lobe Atrophy (MTA) Rating Scale.... In: ResearchGate. https://www.researchgate.net/figure/Sheletens-Medial-Temporal-Lobe-Atrophy-MTA-Rating-Scale-Scoring-was-done-as-follows_fig1_320683190. Accessed Oct 2022

16. Westman E, Cavallin L, Muehlboeck J-S, et al (2011) Sensitivity and Specificity of Medial Temporal Lobe Visual Ratings and Multivariate Regional MRI Classification in Alzheimer's Disease. PLOS ONE 6:e22506. https://doi.org/10.1371/journal.pone.0022506. Accessed Oct 2022

17. Velickaite V, Giedraitis V, Ström K, et al (2017) Cognitive function in very old men does not correlate to biomarkers of Alzheimer's disease. BMC Geriatr 17:1–9. https://bmcgeriatr.biomedcentral.com/articles/10.1186/s12877-017-0601-6. Accessed Oct 2022

18. Mårtensson G, Ferreira D, Cavallin L, et al (2019) AVRA: Automatic visual ratings of atrophy from MRI images using recurrent convolutional neural networks. NeuroImage Clin 23:101872. https://doi.org/10.1016/j.nicl.2019.101872. Accessed Oct 2022

19. Chen Y-C, Tsao H-H, Chu Y-C, et al (2018) Exploring the Spectrum of Subcortical Hyperintensities and Cognitive Decline. J Neuropsychiatry Clin Neurosci 30:130–138. https://doi.org/10.1176/appi.neuropsych.17050099. Accessed Oct 2022

20. Prince M, Wimo A, Guerchet M, et al (2015) World Alzheimer Report 2015. The Global Impact of Dementia. An Analysis of Prevalence, Incidence, Cost and Trends. https://www.alzint.org/u/World-Alzheimer-Report-2021-Chapter-09.pdf. Accessed Oct 2022

21. Sint Lucas-Andreas Hospital, Amsterdam - ppt video online download. https://slideplayer.com/slide/10648481/. Accessed Oct 2022

22. Asselin A, Potvin O, Bouchard L-O, et al (2019) Validation of an Magnetic Resonance Imaging Acquisition and Review Protocol for Alzheimer's Disease and Related Disorders. Can Assoc Radiol J 70:172–180. https://doi.org/10.1016/j.carj.2018.10.008. Accessed Oct 2022

23. Fig. 2 The medial temporal atrophy (MTA) visual rating scale. (a–d)... In: ResearchGate. https://www.researchgate.net/figure/The-medial-temporal-atrophy-MTA-visual-rating-scale-a-d-Coronal-FLAIR-images-showing_fig2_305615685. Accessed Oct 2022

24. Neurology Volume 52, Number 6. In: Neurology. https://www.neurology.org/toc/wnl/52/6. Accessed Oct 2022

25. Themes UFO (2016) Neurodegenerative Diseases and Epilepsy. In: Radiol. Key. https://radiologykey.com/neurodegenerative-diseases-and-epilepsy/. Accessed Oct 2022

26. Figure 1: Medial temporal lobe atrophy scale. In: ResearchGate. https://www.researchgate.net/figure/Medial-temporal-lobe-atrophy-scale_fig3_7583908. Accessed Oct 2022

27. Mårtensson G, Håkansson C, Pereira JB, et al (2020) Medial temporal atrophy in preclinical dementia: Visual and automated assessment during six year follow-up. NeuroImage Clin 27:102310. https://doi.org/10.1016/j.nicl.2020.102310. Accessed Oct 2022

28. Wattjes MP, Henneman WJP, van der Flier WM, et al (2009) Diagnostic imaging of patients in a memory clinic: comparison of MR imaging and 64-detector row CT. Radiology 253:174–183. https://doi.org/10.1148/radiol.2531082262. Accessed Oct 2022

29. Instructional images by Lena Cavallin, Karolinska Huddinge. Accessed October 2022.

30. The Radiology Assistant : Dementia - Role of MRI. https://radiologyassistant.nl/neuroradiology/dementia/role-of-mri. Accessed Oct 2022

31. Håkansson C, Tamaddon A, Andersson H, et al (2021) Inter-modality assessment of medial temporal lobe atrophy in a non-demented population: application of a visual rating scale template across radiologists with varying clinical experience. Eur Radiol 32:. https://doi.org/10.1007/s00330-021-08177-1. Accessed Oct 2022
